# Flexoelectricity Modulated Electron Transport of 2D Indium Oxide

**DOI:** 10.1002/advs.202404272

**Published:** 2024-07-02

**Authors:** Xinyi Hu, Guan Yu Chen, Yange Luan, Tao Tang, Yi Liang, Baiyu Ren, Liguo Chen, Yulong Zhao, Qi Zhang, Dong Huang, Xiao Sun, Yin Fen Cheng, Jian Zhen Ou

**Affiliations:** ^1^ Key Laboratory of Advanced Technologies of Materials Ministry of Education School of Materials Science and Engineering Southwest Jiaotong University Chengdu 610031 China; ^2^ School of Engineering RMIT University Melbourne 3000 Australia; ^3^ School of Mechanical and Electric Engineering Jiangsu Provincial Key Laboratory of Advanced Robotics Soochow University Suzhou 215123 China; ^4^ State Key Laboratory for Manufacturing Systems Engineering School of Mechanical Engineering Xi'an Jiaotong University Xi'an 710049 China; ^5^ Department of Physics The University of Hong Kong Hong Kong 999077 China; ^6^ Inorganic Chemistry University of Koblenz Universitätsstraße 1 56070 Koblenz Germany; ^7^ Institute of Advanced Study Chengdu University Chengdu 610106 China

**Keywords:** 2D indium oxide, electron transport modulation, flexoelectric, stress sensing

## Abstract

The phenomenon of flexoelectricity, wherein mechanical deformation induces alterations in the electron configuration of metal oxides, has emerged as a promising avenue for regulating electron transport. Leveraging this mechanism, stress sensing can be optimized through precise modulation of electron transport. In this study, the electron transport in 2D ultra‐smooth In_2_O_3_ crystals is modulated via flexoelectricity. By subjecting cubic In_2_O_3_ (c‐In_2_O_3_) crystals to significant strain gradients using an atomic force microscope (AFM) tip, the crystal symmetry is broken, resulting in the separation of positive and negative charge centers. Upon applying nano‐scale stress up to 100 nN, the output voltage and power values reach their maximum, e.g. 2.2 mV and 0.2 pW, respectively. The flexoelectric coefficient and flexocoupling coefficient of c‐In_2_O_3_ are determined as ≈0.49 nC m^−1^ and 0.4 V, respectively. More importantly, the sensitivity of the nano‐stress sensor upon c‐In_2_O_3_ flexoelectric effect reaches 20 nN, which is four to six orders smaller than that fabricated with other low dimensional materials based on the piezoresistive, capacitive, and piezoelectric effect. Such a deformation‐induced polarization modulates the band structure of c‐In_2_O_3_, significantly reducing the Schottky barrier height (SBH), thereby regulating its electron transport. This finding highlights the potential of flexoelectricity in enabling high‐performance nano‐stress sensing through precise control of electron transport.

## Graphical abstract

By subjecting 2D cubic In_2_O_3_ crystals to significant strain gradients using an atomic force microscope (AFM) tip, the crystal symmetry is broken, resulting in the separation of positive and negative charge centers. Generated polarization voltage is successfully achieved 20 nN sensitivity of nano‐stress sensing. Also, the flexoelectric coefficient is calculated.

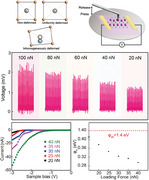

## 1 Introduction

Flexoelectric effect refers to the non‐uniform deformation electromechanical coupling effect of crystals, which breaks the constraint of non‐centrosymmetric crystal structure in piezoelectricity.^[^ ^1^ ^]^ Metal oxides contribute a large number of flexoelectric materials, such as TiO_2_,^[^ ^2^ ^]^ BiFeO_3_,^[^ ^3^ ^]^ and SrTiO_3_.^[^ ^4^ ^]^ When loading stress to the metal oxide crystal surface, a gradient strain forms perpendicularly to the stress direction, switching its crystal structure from centrosymmetric to non‐centrosymmetric.^[^ ^2^, ^5^ ^]^ Under this condition, the positive and negative charge centers of the lattice separate and generate polarization voltage.^[^ ^6^ ^]^ More importantly, the band structure of a flexoelectric crystal is modulated as an electron reconfigured under gradient strain upon its crystal deformation.^[^ ^2^, ^7^ ^]^

In return, such modulation of energy bands also tailors their electron transport of flexoelectric metal oxides.^[^ ^4^, ^8^ ^]^ To enhance the electron transport performance, the energy band that electrons jump from the valence band to the conduction band is supposed to be narrowed to facilitate the separation of positive and negative charges.^[^ ^9^ ^]^ For metal oxides, this electron transition behavior mostly originates from electrons occupied in the highest energy level orbital of metal atoms. Hence, metal atoms with higher energy level electron orbital are preferred for flexoelectric metal oxides. As a post‐transition metal element, 5*s* ^2^ and 5*p_x_* ^1^ electrons configure the outer layer electrons of ground state indium.^[^ ^10^ ^]^ Compared to another anion in the same family, In and O forms tighter bonding due to the large electronegativity differences, leading to a larger strain gradient. When synthesizing cubic‐indium oxides (c‐In_2_O_3_), these three electrons from indium atoms straightforwardly combine with oxide atoms, forming In─O bonds. In this process, the electron from 5*s* ^2^ orbital jumps to the 5*p_y_* ^1^ orbital, indicating this excited state electron in indium oxides could be easily separated from positive charges as a response to the strain gradient.^[^ ^11^ ^]^

To better implement this separation, the strain gradient caused by the stress applied to the c‐In_2_O_3_ crystal surface is supposed to be as large as possible.^[^ ^12^ ^]^ Numerous explorations have been reported to obtain strain gradients. The most commonly used method is bending the substrate where the low‐dimensional metal oxides lay on its surface. Nevertheless, this bending may deform the crystal surface, bringing in the interference of piezoelectricity.^[^ ^13^ ^]^ Moreover, the strain gradient originating from the bending is limited owing to the large deformation area. Alternatively, free‐standing was proposed to eliminate the surface piezoelectric interference.^[^ ^14^ ^]^ However, in practice, the free‐standing nanosheet is not ideally flat. The curvature on the surface due to gravity results in elastic non‐recovery and experimental inaccuracy.^[^ ^15^ ^]^ Additionally, the X‐ray beam is also employed to induce strain.^[^ ^16^ ^]^ Although this method can accurately apply strain, it is non‐preferred in consideration of radiation. To sum up, the challenge to obtain the giant strain gradient is to apply large stress in a micro or nano‐area on the crystal surface. As a consequence, an atomic force microscope (AFM) was employed to enhance the strain gradient.^[^ ^5^, ^17^ ^]^ The AFM tip with an ultra‐small contact area, would be able to induce a local deformation on the In_2_O_3_ crystal lattice. Such deformation can readily approach the order of 10^6^ m^−1^ strain gradient between In_2_O_3_ crystal and substrate.

Herein, a large lateral‐size 2D c‐In_2_O_3_ crystal with an ultra‐smooth and uniform surface was synthesized using the liquid‐metal exfoliation method. The converse flexoelectric performance of c‐In_2_O_3_ crystal was first revealed through piezo‐force microscopy (PFM). Based on its electromechanical response, a dual‐electrodes flexoelectric nano‐stress sensor was fabricated to further investigate the flexoelectric effect of c‐In_2_O_3_. The sensitivity of stress sensing was enhanced to 20 nN, which is four to six orders smaller than other stress sensors. When 100 nN stress was applied to the nano‐stress sensor through the AFM tip, the output voltage and power were generated, achieved their maximum 2.2 mV and 0.2 pW, respectively. By producing a 10^6^ m^−1^ strain gradient through the AFM tip, this nano‐stress sensor enlarged the electrical signal, which overcomes the low electromechanical response of other kinds of stress sensors. According to the output electrical signal, the flexoelectric coefficient and flexocoupling coefficient of c‐In_2_O_3_ were determined as ≈0.49 nC m^−1^ and 0.4 V, respectively. The electron transport variations under increased loading stress by conductive‐atomic force microscopy (C‐AFM) manifest the high flexoelectric polarization of the c‐In_2_O_3_ source from the separation of negative and positive charges under the giant strain gradient.

## 2 Results and Discussion

**Figure 1:**
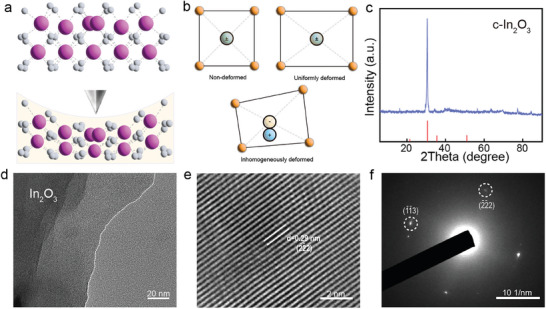
Illustration of the a) Cubic‐In_2_O_3_ (c‐In_2_O_3_) crystal structure deformation with a tip‐induced inhomogeneous strain, and b) the positive and negative charges separate upon the inhomogeneous deformation of c‐In_2_O_3_ crystal. c) XRD pattern of c‐In_2_O_3_ nanosheet, indicating the high crystallinity of crystal. d–f) Low magnification TEM image, HRTEM image and its corresponding SAED of the c‐In_2_O_3_ nanosheet.

The indium oxide nanosheet was obtained through a liquid metal exfoliation process. Under a controlled Cabrera‐Mott oxidation condition, a uniform metal oxidation surface was formed which could be directly printed onto the substrate through van der Waals (vdW) force (Figure S1, Supporting Information).^[^ ^18^ ^]^The In_2_O_3_ nanosheet featured a highly smooth and large lateral size appearance (Figure S2, Supporting Information). From the AFM image, the nanosheet has a lateral size of over 30 µm and a thickness of 4.8 nm, corresponding to four unit cells (Figure S3, Supporting Information). The XPS spectrum of In 3*d* was deconvoluted into two peaks at the binding energies of 452.6 and 444.9 eV, assigned to the 3*d_5/2_* and 3*d_3/2_* energy states, respectively (Figure S4, Supporting Information).^[^ ^19^ ^]^ The high‐intensity peak of (‐2 ‐2 2) plane in the X‐ray diffraction (XRD) pattern indicates the synthesized c‐In_2_O_3_ nanosheet owns a high crystallinity shown in **Figure** **1**c (PDF#88‐2160).^[^ ^20^ ^]^The microstructure derived from high‐resolution transmission electron microscope (HRTEM) image proves the synthesized c‐In_2_O_3_ nanosheet possesses a *d*‐space of ≈0.29 nm, matching the (2_2_2) plane of c‐In_2_O_3_ (Figure 1e). The selected area electron diffraction (SAED) image confirms the In_2_O_3_ nanosheet belongs to the Ia‐3 space group, which is centrosymmetric (Figure 1f).^[^ ^21^ ^]^

**Figure 2:**
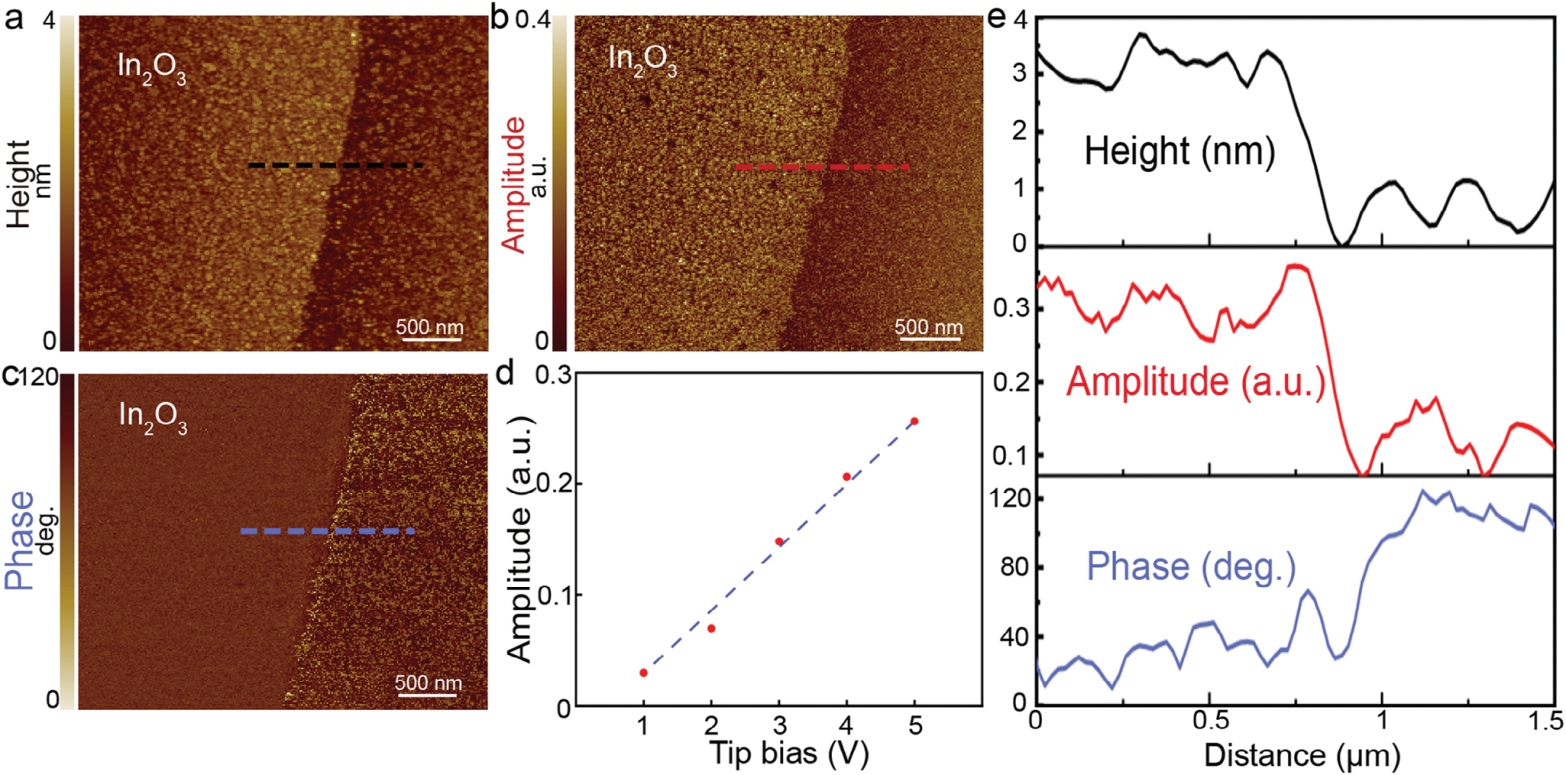
PFM topography a), amplitude response b), and phase response c) of a c‐In_2_O_3_ nanosheet. d) Amplitude variations under different tip biases with the 0 V sample bias. e) Line profiles of topography, amplitude, and phase corresponding to their corresponding lines in (a–c). The c‐In_2_O_3_ nanosheet exhibits a thickness of ≈2.6 nm, which matches the thickness of two unit cells.

As the flexoelectricity effect is a strain‐electric coupling effect, the stress‐electric field relation could be expressed as Equation (1):

(1) $$\begin{array}{c} \sigma \;=\;\mu \nabla E \end{array}$$

where σ is the stress, ∇*E* is the electric field, and *µ* is the flexoelectric coefficient.^[^ ^22^ ^]^ According to Equation (1), the centrosymmetric c‐In_2_O_3_ nanosheet proceeds a strain response to a gradient electric field. To ensure the contacts, minimal compressive stress is preferred to apply to c‐In_2_O_3_ nanosheet through a nano‐scale conductive AFM tip.^[^ ^5^, ^23^ ^]^ As a consequence, an electric field gradient is generated and decayed with the distance away from the contact area when applying a tip bias to the nanocrystal. In this circumstance, the converse flexoelectric effect of the c‐In_2_O_3_ nanosheet is reflected in the amplitude in PFM mode, which represents the displacement of the tip.^[^ ^24^ ^]^ Meanwhile, the phase refers to the direction of the deflection. The PFM topology, amplitude, and phase response under 5 V bias are demonstrated in **Figure** **2**a–c, respectively. The measured c‐In_2_O_3_ nanosheet is ≈2.6 nm (Figure 2a), which is two unit‐cell thick, excluding the possibility of odd layer symmetry change in the atomic scale nanosheet. The amplitude and phase (Figure 2b,c) mappings clearly showed a strong flexoelectric response (Figure 2d). With the increase of the bias, the electric field gradient generated through the AFM tip was enhanced, along with a remarkable upward trend of flexoelectronic response (Figure S6, Supporting Information).

**Figure 3:**
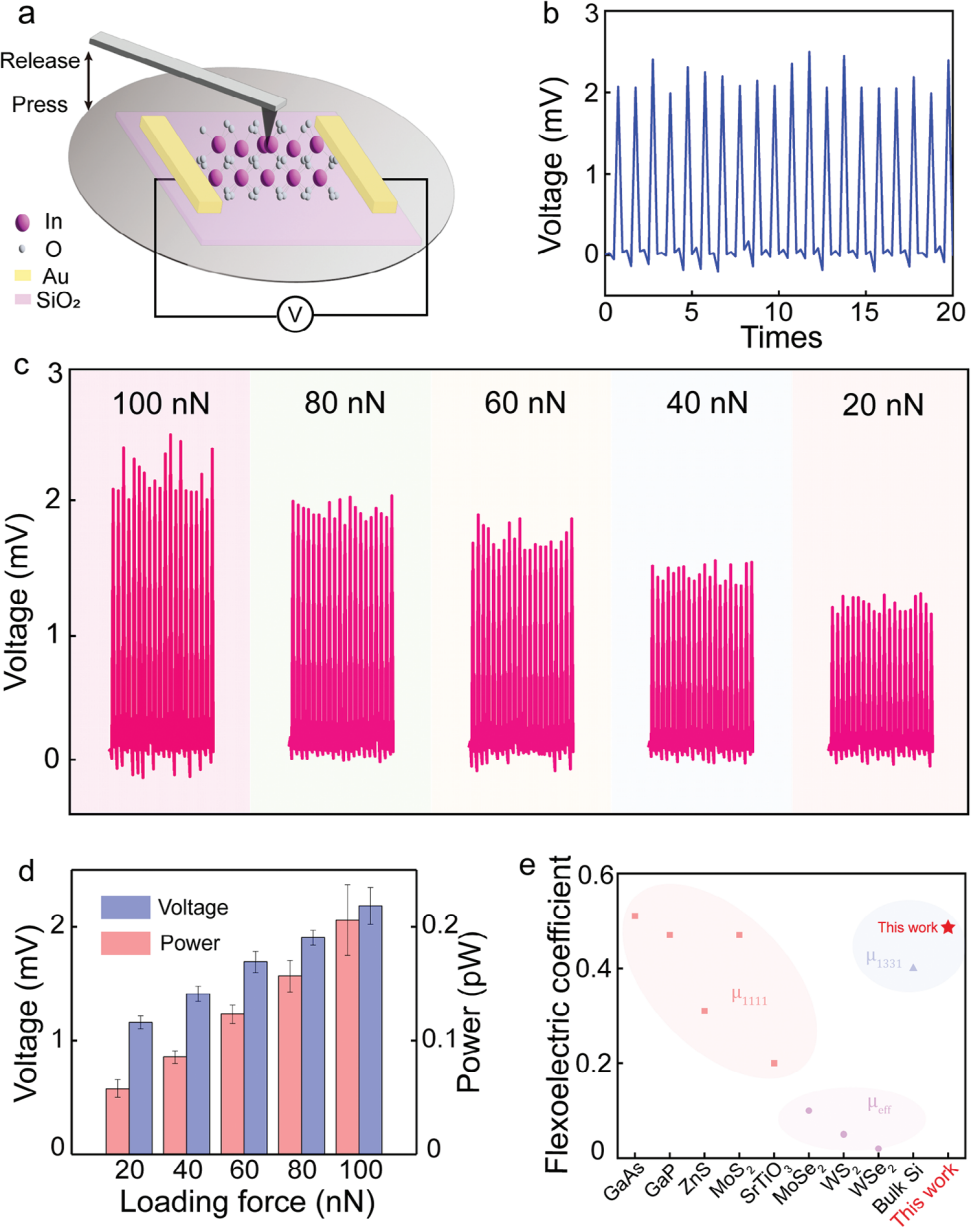
The measurement of the developed nano‐strain sensor fabricated based on c‐In_2_O_3_ nanosheet. a) Schematic illustration of the measurement setup. b) The sensor voltage output curve repeated 20 times under 100 nN loading stress and c) under 100–20 nN loading stress with a degradation of 20 nN. d) The output of the nano‐strain sensor. e) Flexoelectric coefficient comparison between reported flexoelectricity materials and the c‐In_2_O_3_ nanosheet.

As the great potential of the c‐In_2_O_3_ in flexoelectric effect from PFM mappings, the dual‐electrode nano‐stress sensor was fabricated on the SiO_2_/Si substrate using a liquid metal process. The configuration and schematic of the sensor characterization are depicted in **Figure** **3**a and the setup of nano‐stress sensor measurements are depicted in Figure S10 (Supporting Information). Generally, the polarization is derived from the centrosymmetric broken caused by the strain gradient, leading to a conversion from mechanical movement to electrical signal. To precisely generate such a giant strain gradient, a nano‐stress loading gauge was designed based on the AFM system. To exclude the triboelectric effect, the pulse nano‐strain was applied to the c‐In_2_O_3_ nanosheet reciprocally through the AFM nano‐tip. From Figure 3b, a maximum of 2.2 mV output voltage was achieved under 100 nN loading stress. Furthermore, along with the loading stress decrease from 100 to 20 nN, the responsive output voltage exhibits a degradation from 2.2 to 1.2 mV due to the flexoelectronic effect. As shown in Figure 3d, the output voltage and power are enhanced nonlinearly as loading stress increases, which is further supported by Figure S12 (Supporting Information). The nano‐stress sensor has a distinct output voltage due to the bond deformation caused by the flexoelectricity effect, enlarging the response electric signal. Therefore, the experimental stress sensing sensitivity achieved 20 nN, which is four to six orders smaller than other stress sensors fabricated with low dimensional materials based on piezoresistive, capacitive, and piezoelectric effects (**Table** **1**). Besides, for other kinds of reported stress sensors based on the piezoresistive, capacitive, and piezoelectric effect, are in millimeter or micrometer size to ensure structural strength. Therefore, the stress is applied to a large area which also constrains their stress sensitivity. The polarization and flexoelectric coefficient relation is introduced to better interpret its electromechanical responsive process, as follows:

(2) $$\begin{array}{c} \;P_{i}=\mu_{\mathrm{ijkl}}\;\frac{\partial S_{\mathrm{jk}}}{\partial x_{l}} \end{array}$$

where *P* is the flexoelectric induced polarization, µ is the flexoelectric coefficient, a fourth‐rank tensor, *S* is elastic strain, and *x* indicates the direction distance, the $\frac{\partial S_{jk}}{\partial x_{l}}$ represents the strain gradient.^[^ ^25^ ^]^ As described previously, the tip‐sample contact model could be elucidated using the Hertz‐Contact model for stain gradient (details shown in Note S1, Supporting Information).^[^ ^26^ ^]^ Compared to the reported flexible substrates, the hard substrate provides more possibilities for a giant strain gradient (≈10^6^ m^−1^). As mentioned above, the In_2_O_3_ nanosheet has a crystallographic structure of the Ia‐3 space group, the non‐zero independent element of the strain and flexoelectric coefficient can be expressed using a matrix in Note S2 (Supporting Information). The polarization of the c‐In_2_O_3_ is reflected on the sensor output voltage, which can be expressed by the following equations:

(3) $$\vec{P}=\left(\varepsilon -\varepsilon_{0}\right)\;\cdot \vec{E}$$

(4) $$\begin{array}{c} P\;=\;\varepsilon f_{\mathrm{coupling}}\left(\frac{\partial u_{t}}{\partial x_{l}}\right) \end{array}$$

**Table 1:** Comparison of stress sensors fabricated with other low dimensional materials.

Materials	Measurement mechanism	Sensitivity [nN]	References
Graphene/Ag nano‐wires	Piezoresistive	1 × 10^6^	[29]
Ag nano‐wires	Capacitive	4 × 10^5^	[30]
CNT	Capacitive	3.6 × 10^6^	[31]
Graphene	Capacitive	5 × 10^7^	[32]
CNT doped ceramics film	Piezoelectric	4 × 10^7^	[33]
PbTiO_3_ nano‐wire/Graphene	Piezoelectric	1 × 10^7^	[34]
This work	Flexoelectric	20	–

The built‐in electric field can be calculated by the $E\;=\frac{V}{d}\;$, where *d* is the nanosheet thickness and *V* is the output voltage measured above by the source meter, *f_coupling_* refers to the flexocoupling coefficient.^[^ ^1^, ^27^ ^]^ By the model described above, the flexoelectric coefficient µ_1331_ of c‐In_2_O_3_ nanosheet is calculated to be ≈0.49 nC m^−1^ and the flexocoupling coefficient *f_coupling_* ≈0.4 V. Although the flexoelectric coefficient of c‐In_2_O_3_ is not outstanding when compared to the piezoelectric material such as BaTiO_3_.^[^ ^28^ ^]^ However, it is relatively high referring to the reported 2D materials (Figure 3e). Due to the tight bonding between metal and chalcogens atoms of 2D TMDCs, the in‐plane strain gradient is considerably larger than the strain gradient generated from out‐of‐plane under the same stress due to its weak interlayer vdW force. As a consequence, the large flexoelectric coefficient comes from the ionic bonding of indium and oxygen and the large lattice parameters result in a giant strain gradient. The output voltage of the sensor exhibits a strong thickness dependence due to the electronic transport difference (Figure S14, Supporting Information). With the thickness increasing, the electron transport decreases, reflecting in decrease of the output voltage. Additionally, to examine the influence of the electrodes, we fabricated new sensors using Pt electrodes. The optical image of the sensor with Pt electrodes was shown in Figure S15 (Supporting Information). The output voltage of the sensors with Pt electrodes shows no significant difference compared to the Au electrodes in Figure S16 (Supporting Information).

**Figure 4:**
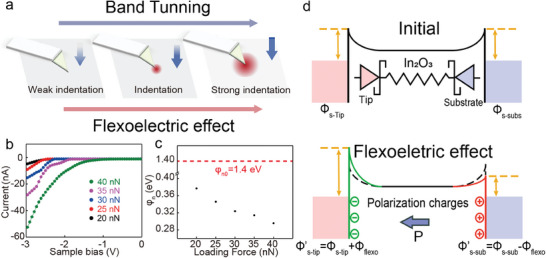
a) Schematic illustration of the measurement under different force indentations. b) Current–voltage curves derived from C‐AFM measurement of c‐In_2_O_3_ nanosheet under different loading stress. The loading stress is precisely applied to the nanosheet through an AFM tip with a platform controller. c) Calculated SBH φ_*n*_ under different loading stress. The red dash line is the initial SBH φ_*n*0_. d) Schematic illustration of flexoelectric induced band regulation of initial, positive flexoelectric potential and negative flexoelectric potential state. The arrow lable indicates the polarization direction.

The nanoindentation not only generates the output voltage of the sensor but also forms a built‐in inner‐crystal polarization of the nanosheet. The inner‐crystal polarization will arouse a built‐in electric field, significantly influencing the concentration and distribution of free carriers at the metal–semiconductor (M–S) interface.^[^ ^2^, ^35^ ^]^ The negative flexoelectric potential induced on the semiconductor repels electrons and attracts holes at the M–S contact interfaces, resulting in a band upward bending. Further, the bending band alters the Schottky barrier height (SBH) and finally reflects on the electron transport current.^[^ ^36^ ^]^ C‐AFM based on contact was performed to characterize electrical migration properties under different nanoindentation. **Figure** **4**d shows the influence of the Schottky contact modulation by flexoelectric polarization. Under the assumption of the above theory, the tip‐induced direct‐flexoelectronic effect can effectively regulate the interface SBH and width, thereby controlling the charge transport. Figure 4b shows the *I–V* curve under the different loading stress. The current increase with the ascending loading stress can be attributed to the flexoelectric effect induced by the electron transport modulation. Here, the classical thermal emission theory is introduced to describe the current and SBH relation:

(5) $$\begin{array}{c} I\;=A^{*}\;AT^{2}\exp\left(-e\varphi_{n}/\mathrm{kT}\right) \end{array}$$

Here A* is the Richardson constant, A*= 4πekm*/*h* ^3^, *e* is the elementary charge, *k* is the Boltzmann constant, m* is the effective mass, and *h* is the Planck constant. *A* is the contact area; *T* is the temperature and here we choose T  =  300 K, the room temperature as the experiment temperature; φ_*n*_ is the total effective barrier height, φ_*n*_ = φ_*n*0_  + φ_*flexo*_, φ_*n*0_ denotes to initial SBH between the nanosheet and metal, and φ_*flexo*_ refers to the flexoelectric effect‐induced potential. The initial SBH can be expressed as φ_*n*0_ = φ_*s*_  − χ, here φ_*s*_ is the work function of the substrate metal, and χ is the electron affinity.^[^ ^2^, ^8^, ^36^ ^]^ The initial band structure of c‐In_2_O_3_ nanosheet was measured by scanning tunneling spectroscopy (STS). Figure S9 (Supporting Information) indicates the In_2_O_3_ nanosheet is the n‐type, with the bandgap of ≈2.6 eV. The arrow indicated the valence band (VB), fermi level (FL), and conduction band (CB) of the nanosheet, respectively.^[^ ^37^ ^]^ The initial work function of the nanosheet and substrate was measured by the Kelvin probe force microscopy (KPFM) shown in Figure S8 (Supporting Information), which was ≈4.2 eV.^[^ ^38^ ^]^ Thus, the initial SBH φ_*n*0_ is ≈1.4 eV shown in Figure 4c. The red dash line indicated the initial SBH and the black plot was the total SBH, the reduction barrier height is the flexoelectric effect induced barrier modulation. As the tip force increased, the total barrier height showed a decreasing trend, which agreed with the barrier height and polarization direction (Figure 4d).

## 3 Conclusion

In conclusion, the large lateral size ultra‐smooth 2D c‐In_2_O_3_ nanocrystal possessed a high crystallinity with a centrosymmetric la‐3 space group. The amplitude and phase mappings obtained from PFM clearly exhibited a strong flexoelectric response, verifying the converse flexoelectric effect of In_2_O_3_ nanocrystal. A dual‐electrodes flexoelectric nano‐stress sensor was introduced to further investigate the flexoelectric effect of c‐In_2_O_3_. The output voltage and power reached their maximum 2.2 mV and 0.2 pW when applying a 100 nN force to the c‐In_2_O_3_ lattice through the AFM tip. Such a nano‐stress sensor enlarged the electrical signal through a giant strain gradient by AFM tips. Due to the flexoelectric effect, the sensitivity reached an impressive value, i.e., 20 nN, which is four to six orders smaller than other stress sensors fabricated based on piezoresistive, capacitive, and piezoelectric effects. The flexoelectric coefficient and flexocoupling coefficient of c‐In_2_O_3_ were determined as ≈0.49 nC m^−1^ and 0.4 V, respectively. The electron transport variations indicating the flexoelectric effect‐induced polarization regulate the M–S contact SBH, affiliating the separation of negative and positive charges under the giant strain gradient. This work first studies the flexoelectric behavior of 2D c‐In_2_O_3_ nanocrystal, determines the flexoelectric coefficient and flexocoupling coefficient, and the nano‐stress sensor has been fabricated. Through the effect, the electron transport properties of c‐In_2_O_3_ is successfully tailored, paving its way to electromechanical nano sensing.

## 4 Experimental Section

### Synthesis of In_2_O_3_ Nanosheet

The In_2_O_3_ nanosheet was prepared by liquid metal van der Waals exfoliation technique. The whole reaction process was in a glove box (Miqi Equipment Co., Ltd, Changsha, China), in a low oxygen condition of 0.5%, with nitrogen as the protective gas. The substrate and pure indium metal (Shengshida Metal Materials Co., Ltd) was placed on the hotplate at 200 °C. To obtain the high‐quality uniform nanosheet, the surface impurity was removed, and the exposed pure liquid in was oxidized for 30 s under the conditions mentioned above. After oxidation, printing the droplet onto the SiO_2_/Si (300 nm/500 µm) substrate. Typically, the oxygen concentration and the oxidation time mainly affect the thickness of the nanosheet and the printing force was significantly influences on the quality of the nanosheet.

### Characterization of the In_2_O_3_ Nanosheet

Scanning electron microscopy and energy dispersive spectroscopy (SEM & EDS, FEI Inspect F50) were used to characterize the surface morphologies and corresponding chemical elements. X‐ray photoelectron spectroscopy (XPS, K‐Alpha, Al Kα excitation source) was utilized to determine the chemical compositions. The crystal structure was confirmed by the X‐ray diffractometer (XRD, PANalytical, The Netherlands, Cu Kα radiation source). TEM, HRTEM, and SAED images were captured by JEOL‐2100F with a Gatan Orius SC1000 CCD camera and Phillips CM200, Netherlands. The sample was directly printed on the copper‐carbon TEM grids under the same condition mentioned above. The atomic force microscopy (AFM, Park system NX10, Korean) was used for nanosheet morphology investigation. STS was performed to obtain the band structure information of the nanosheet. STS spectra were shown in Figure S9 (Supporting Information) with a bandgap of ≈2.6 eV and a tunneling current set point of 0.5 nA.

### PFM Measurement

The piezo‐force microscopy (PFM) measurement was conducted on Park AFM system (Park system NX10, Korean). The vertical piezo‐force microscopy mode was employed to reveal the piezoelectric performance of the nanosheet. The nanosheet was printed onto a conductive substrate (Pt/Cr/SiO_2_/Si:100 nm/30 nm/300 nm/500 µm); the substrate was pasted on a sample disk with low‐temperature cured silver paste. Before loading the sample in the measurement, the sample was blown for 30 min using an ionic fan to remove surface charge. The PPP‐EFM conductive tip (Pt/Ir coated, 2.8 N m^−1^ spring constant) was used to conduct the measurement by applying different tip bias with an AC driving voltage at the frequency of 17 kHz.

### Nano Strain Sensor Measurement

The nano strain sensor measurement was utilized by the Park AFM system (Park system NX10, Korean) and source meter (Keithley SMU 2602b). The nanosheet was printed onto a conductive substrate (SiO_2_/Si: 300 nm/500 µm); the dual pad electrodes (Au/Ti: 100 nm/10 nm) were prepared by the ebeam evaporation process. The AC‐160TS silicon tip (7 nm tip radius, 26 N m^−1^ spring constant) was used to conduct the measurement by applying 20–100 nN loading force.

## Conflict of Interest

The authors declare no conflict of interest.

## Supporting information

Supporting Information

## Data Availability

Research data are not shared.
