# Supplementary material for: Flexoelectricity Modulated Electron Transport of 2D Indium Oxide
Source: Adv Sci (Weinh). 2024 Jul 2;11(33):2404272. doi: 10.1002/advs.202404272 (PMC11434226; doi:10.1002/advs.202404272)
Supplement: Supplementary file 1 — Supporting Information [file ADVS-11-2404272-s001.docx]

Supporting Information

Xinyi Hu, Guan Yu Chen*, Yange Luan, Tao Tang, Yi Liang, Baiyu Ren, Liguo Chen, Yulong Zhao, Qi Zhang, Dong Huang, Xiao Sun *, Yin Fen Cheng, Jian Zhen Ou*.

X. Hu, Dr. G. Y. Chen, T. Tang, Y. Liang, Dr. B. Ren, Prof. J. Z. Ou

Key Laboratory of Advanced Technologies of Materials, Ministry of Education, School of Materials Science and Engineering, Southwest Jiaotong University, Chengdu 610031, China

[guanyu.chen@swjtu.edu.cn](mailto:guanyu.chen@swjtu.edu.cn), jzou@swjtu.edu.cn

Y. Luan, Prof J. Z. Ou

School of Engineering, RMIT University, Melbourne 3000, Australia
jianzhen.ou@rmit.edu.au

Prof L. Chen

School of Mechanical and Electric Engineering Jiangsu Provincial Key Laboratory of Advanced Robotics, Soochow University, Suzhou 215123, China

Prof Y. Zhao, Dr. Q. Zhang

State Key Laboratory for Manufacturing Systems Engineering, School of Mechanical Engineering, Xi'an Jiaotong University, Xi'an 710049, China

Dr. D. Huang

Department of Physics, the University of Hong Kong, Hong Kong 999077, China

Dr. X. Sun

Inorganic Chemistry, University of Koblenz, Universitätsstraße 1, 56070 Koblenz, Germany

Xiaosun@uni-koblenz.de

Dr. Y. F. Cheng

Institute of Advanced Study, Chengdu University, Chengdu 610106, China

**Note S1**

At the surface, the normal stress distribution (as a function of the distance from the contact center) is described by the Hertz contact mechanics of the spherical indenter, as follows:

$$\sigma_{33}\left( r \right)=\left\{ \begin{aligned} -\frac{3F}{2\pi a^{2}}\sqrt{1-\frac{r^{2}}{a^{2}}}, r\leq a \\ 0, r\geq a \end{aligned} \right.$$

Where a=$\sqrt[3]{\frac{3}{4}\frac{FR}{E_{eff}}}$ is the contact radius determined by contact force $F$, tip radius $R$, and effective contact Young’s modulus $E_{c}$ *.* The nanosheet and tip contacted effective Young’s modulus is given by $\frac{\boldsymbol{1}}{\boldsymbol{E}_{\boldsymbol{C}}}\boldsymbol{=}\frac{\left( \boldsymbol{1-}\boldsymbol{v}_{\boldsymbol{s}}^{\boldsymbol{2}} \right)}{\boldsymbol{E}_{\boldsymbol{S}}}\boldsymbol{+}\frac{\boldsymbol{(1-}\boldsymbol{v}_{\boldsymbol{tip}}^{\boldsymbol{2}}\boldsymbol{)}}{\boldsymbol{E}_{\boldsymbol{tip}}}$***_,_*** where $E_{C}$ and $v$ are Young’s modulus and Poisson ratio respectively. In this work, the tip and In_2_O_3_ Young’s modulus are 160 GPa^[1]^ and 140 GPa^[2]^, respectively. Since the Young's modulus of the probe is greater than that of In_2_O_3_, thus the probe is rigid. At the contact interfaces, assuming that the displacement is continuous and the tip-nanosheet contact depth under loading stress $F$ is $h=\left( \frac{3F}{4E_{c}\sqrt{R}} \right)^{\frac{2}{3}}$. The strain related to stress can be expressed as $u_{33}=\frac{\sigma_{33}}{E_{C}}.$

The model we build assumes that the tip is ideally rigid. However, in practical, the tip will not be ideally rigid during the repeated loading because of the hard contact with the sample. The obvious blunt effect of AFM tip will appear along with the repeat of loading over 100 times.

This phenomenon only occurs during a large number of repeated uses. Due to this phenomenon, the tip was replaced intermittently (60-70 times) to ensure the accuracy of the measurement results.

**Note S2 Theoretical calculation of direct flexoelectric coefficient**

As the contact model we constructed, the relationship between polarization, direct flexoelectric coefficient and strain gradient can be expressed as:

$$P_{i}=\mu_{ijkl}\frac{\partial\varepsilon_{jk}}{\partial x_{l}}$$

Where, $P_{i}$ is the polarization; $\mu_{ijkl}$ is the flexoelectric coefficient, a forth rank tensor; $\varepsilon_{jk}$ is elastic strain; and $x_{l}$ is the axis.

For c-In_2_O_3_, according to the cubic symmetry, the direct flexoelectric coefficient contains three independent coefficients, $\mu_{1111}=\mu_{2222}=\mu_{3333}, \mu_{1122}=\mu_{1133}=\mu_{2211}=\mu_{2233}=\mu_{3311}=\mu_{3322},$and $\mu_{1221}=\mu_{1331}=\mu_{2112}=\mu_{2332}=\mu_{3113}=\mu_{3223}$. The matrix can be expressed as following:

$$\left[ \begin{matrix} \mu_{11} & 0 & 0 & \mu_{14} & 0 & 0 & \mu_{14} & 0 & 0 & 0 & \mu_{111} & 0 & 0 & 0 & \mu_{111} & 0 & 0 & 0 \\ 0 & \mu_{14} & 0 & 0 & \mu_{11} & 0 & 0 & \mu_{14} & 0 & \mu_{111} & 0 & 0 & 0 & 0 & 0 & 0 & 0 & \mu_{111} \\ 0 & 0 & \mu_{14} & 0 & 0 & \mu_{14} & 0 & 0 & \mu_{11} & 0 & 0 & 0 & 0 & 0 & 0 & 0 & \mu_{111} & 0 \end{matrix} \right]$$

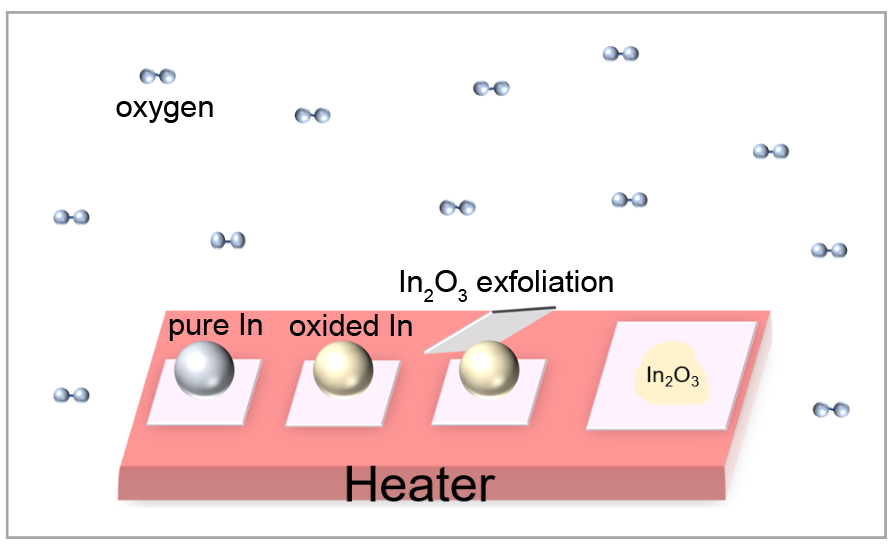


**Figure S1.** The schematic of liquid metal exfoliation process. The pure indium was molten and reacted with low concentration oxygen, forming an ultra-thin In_2_O_3_ nanosheet. The In_2_O_3_ nanosheet was exfoliated using the substrate printing method.


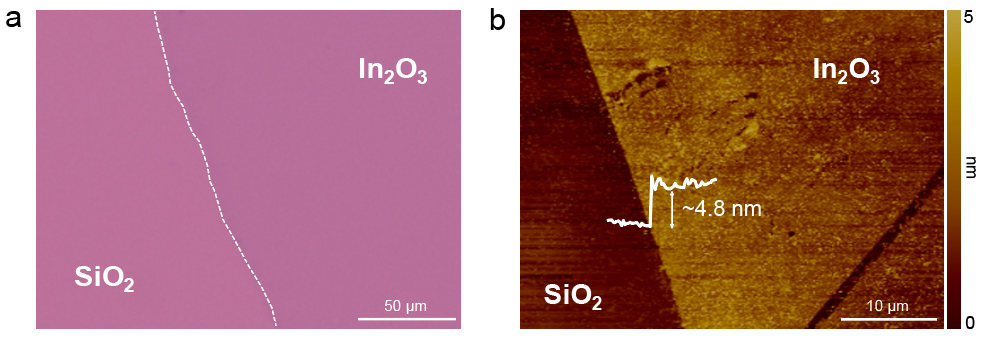


**Figure S2.** a) Optical image and b) AFM mapping of a c-In_2_O_3_ nanosheet. The insertion is its thickness profile.


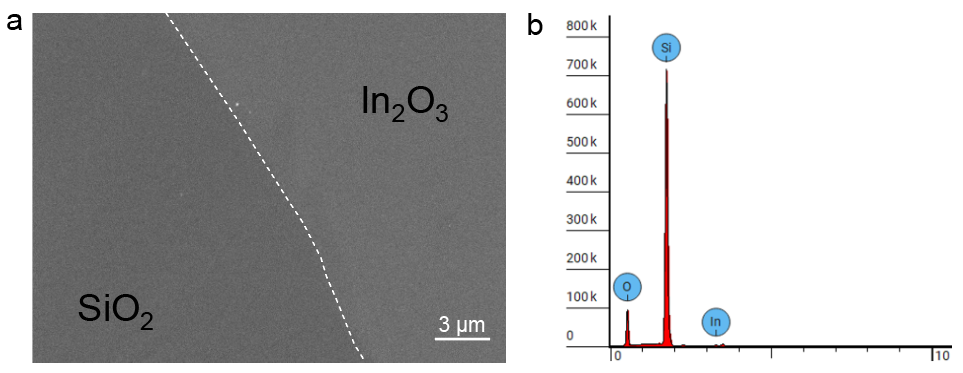


**Figure S3.** a) SEM image of the In_2_O_3_ nanosheet. b) EDS result indicates the chemical elements of nanosheet are In and O.


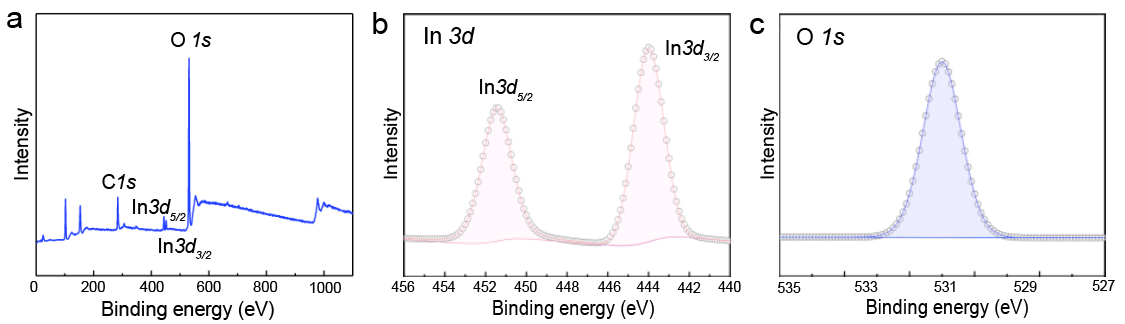


**Figure S4.** **XPS spectra of the In_2_O_3_ nanosheet.** a) Survey spectrum indicates the chemical states are O *1s*, In 3*d_3/2_*, and In 3*d_5/2_*, located at 531.2 eV, 452.6 eV and 444.9 eV, respectively. b) The spectrum of In *3d*. c) The spectrum of O *1s*.


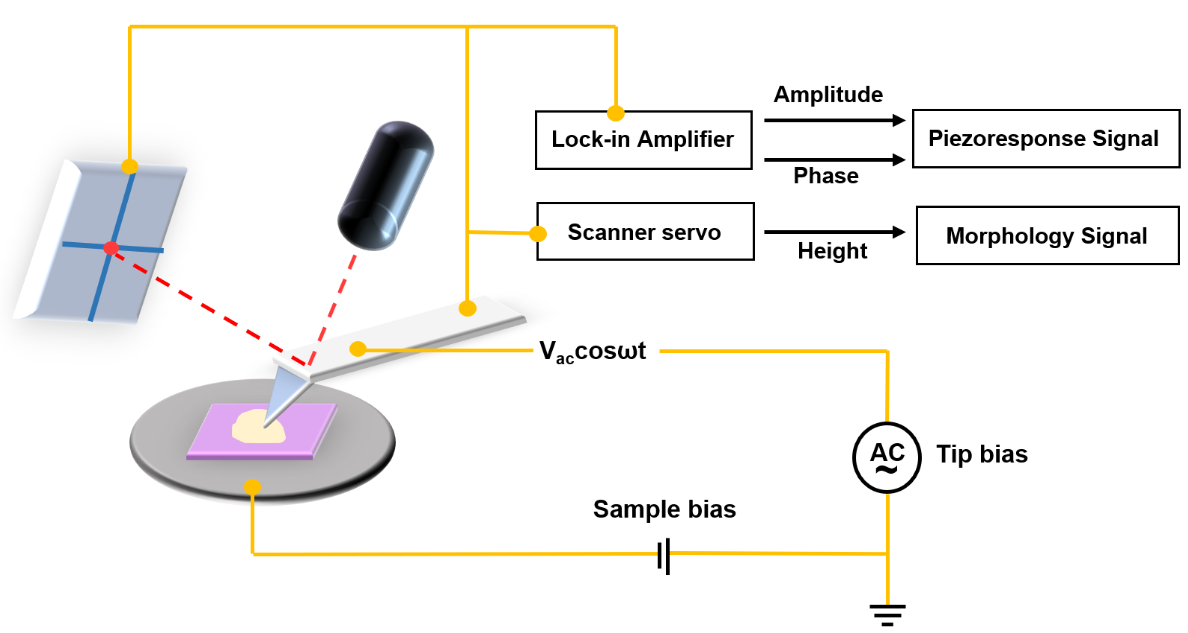


**Figure S5.** The schematic of PFM measurement mechanism.


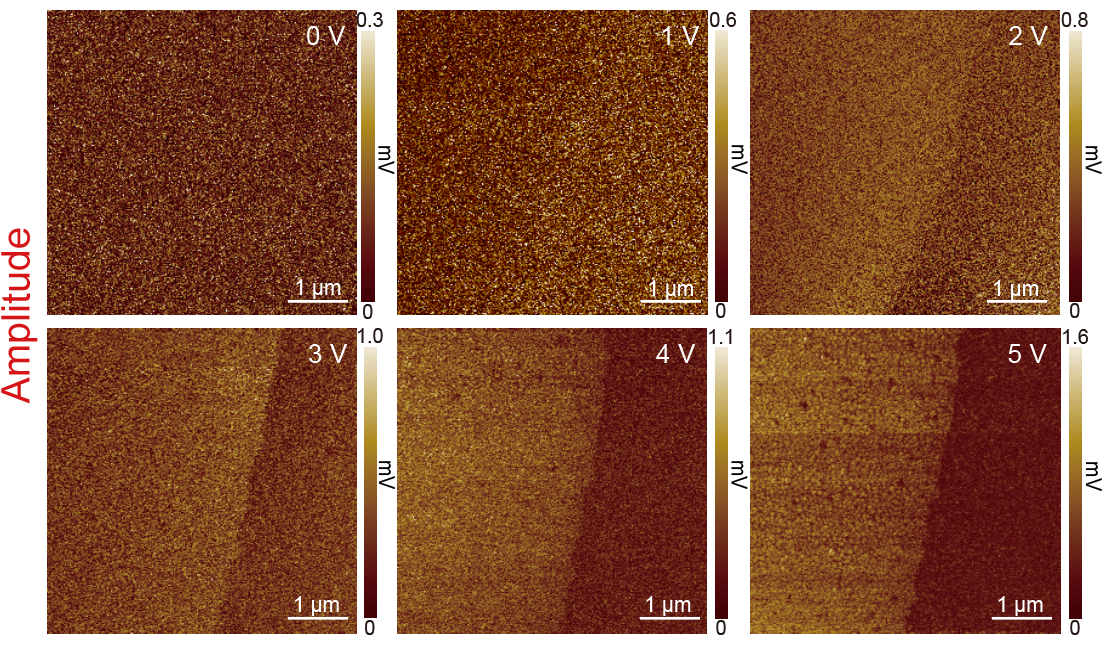


**Figure S6.** PFM amplitude images of the In_2_O_3_ nanosheet. The tip bias increased from 0-5 V.


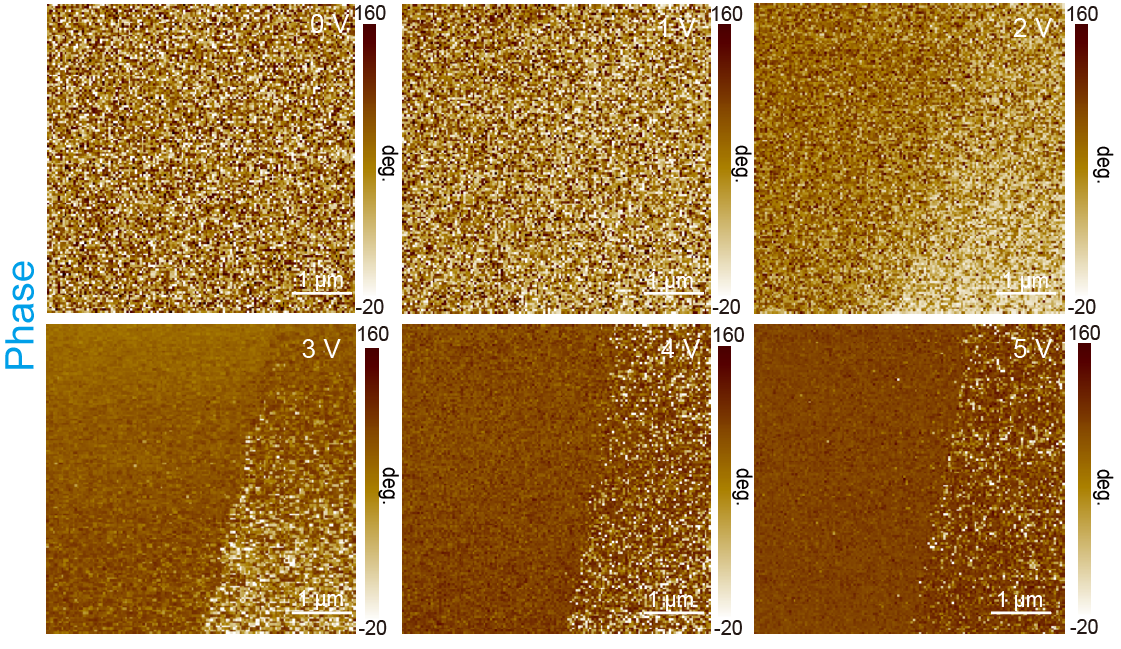


**Figure S7.** PFM phase images of the In_2_O_3_ nanosheet. The tip bias increased from 0-5 V.


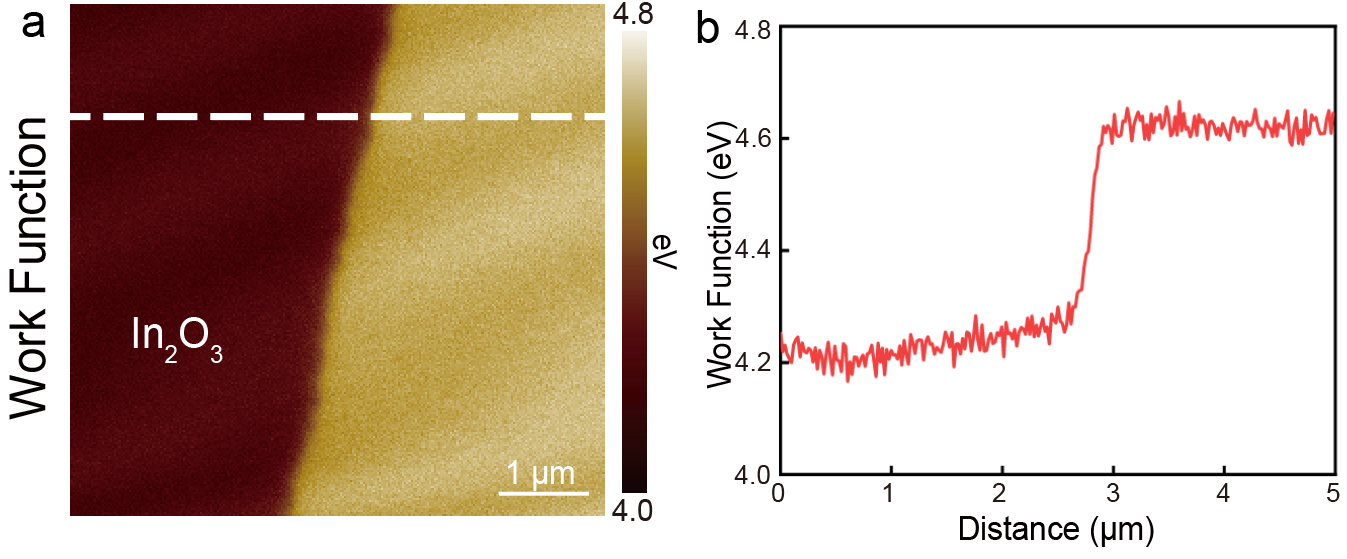


**Figure S8.** The KPFM measurement of the In_2_O_3_ nanosheet. The image should the work function shows a clear difference between the sample and substrate. Line profile indicated the work function of the In_2_O_3_ nanosheet is 4.2 eV.


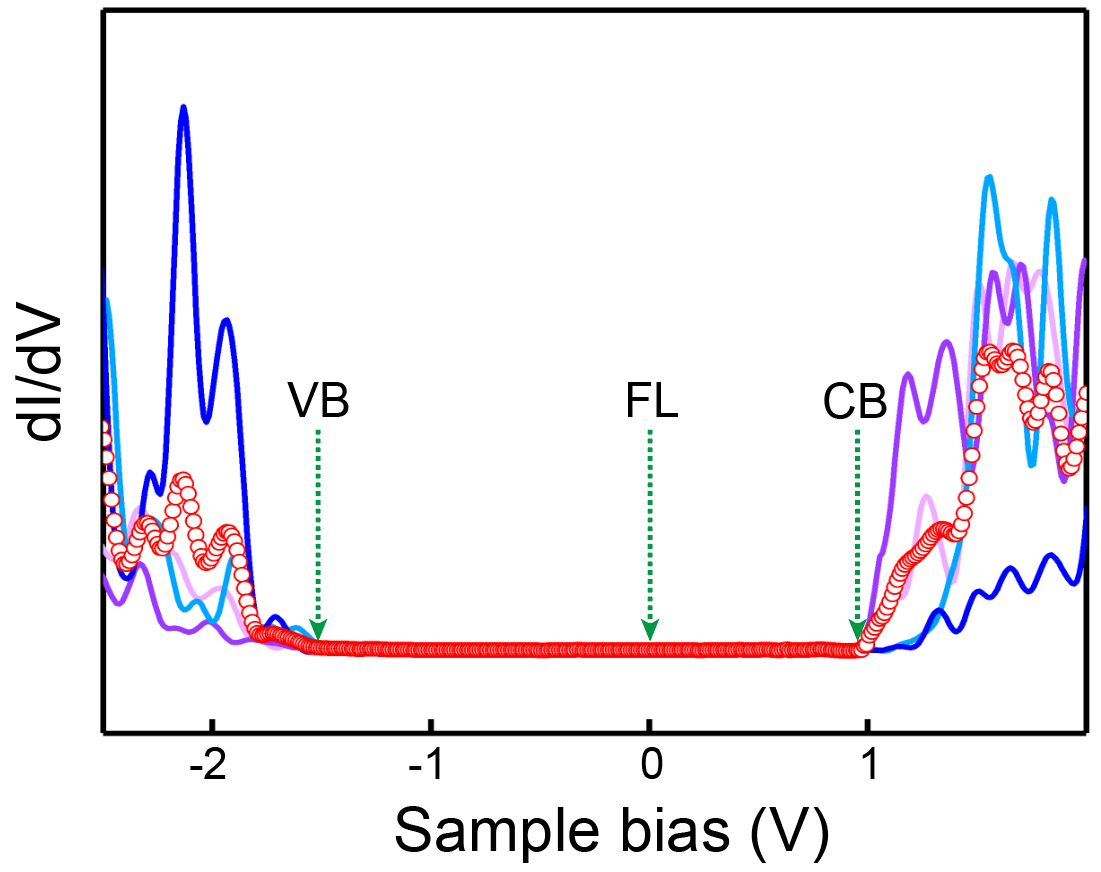


**Figure S9.** STS spectra of the In_2_O_3_ nanosheet. Three green arrows from left to right represents VB, FL and CB, respectively.

**
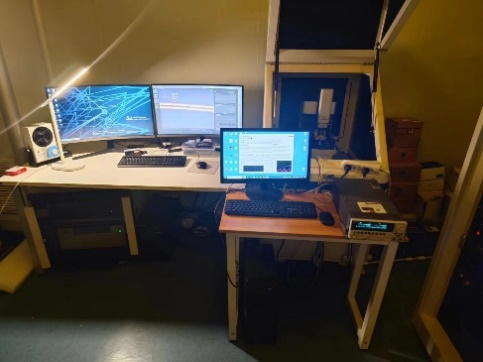

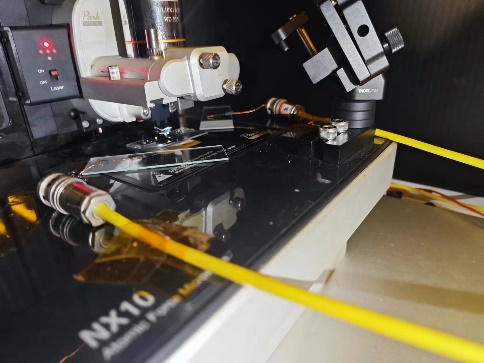
**

**Figure S10.** Setup of the flexoelectric nano-strain sensor measurements**.**


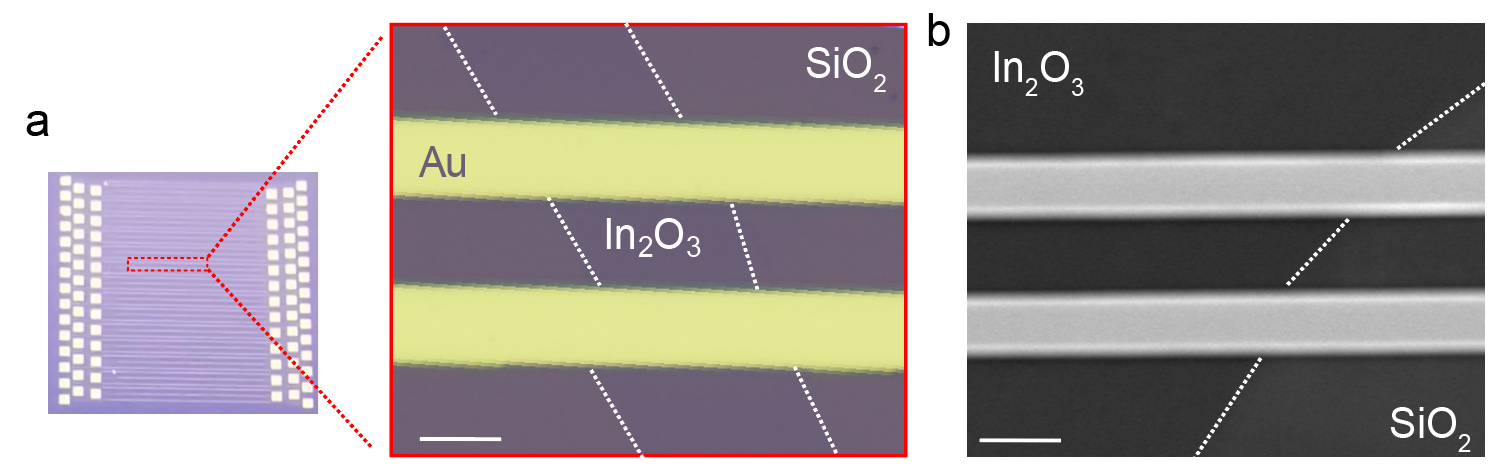


**Figure S11.** Flexoelectric nano-strain sensor images**.** a) Optical images of the sensor with the scale bar 7 μm. The contact pads are 0.5 х 0.5 mm^2^, and dual Au electrodes distance are 7 μm. b) SEM image of the device with the scale bar 5 μm.


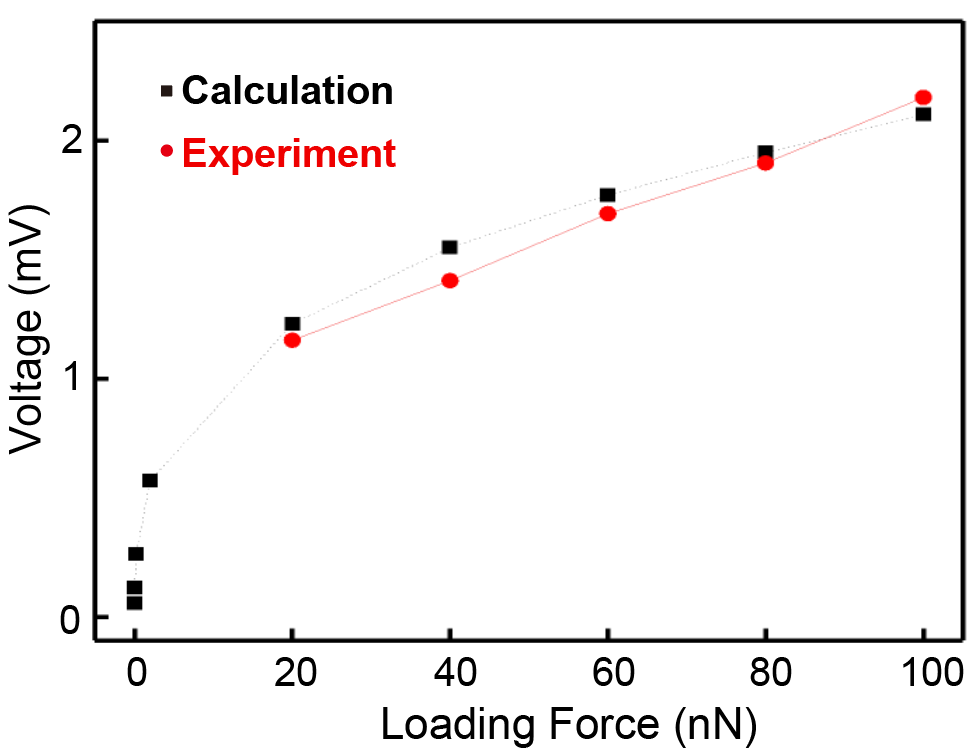


**Figure S12.** The experimental and theoretical output of the device.


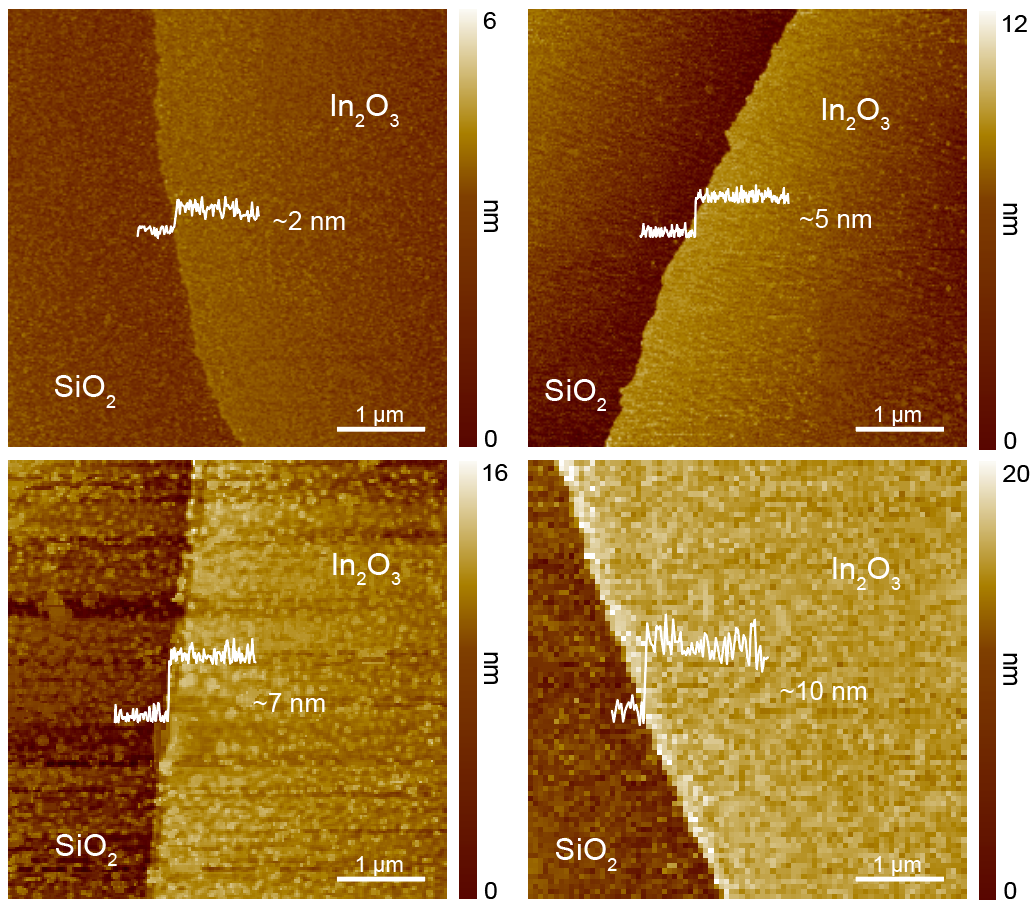


**Figure S13.** The AFM images of c-In_2_O_3_ with different thicknesses for nano-stress sensing.


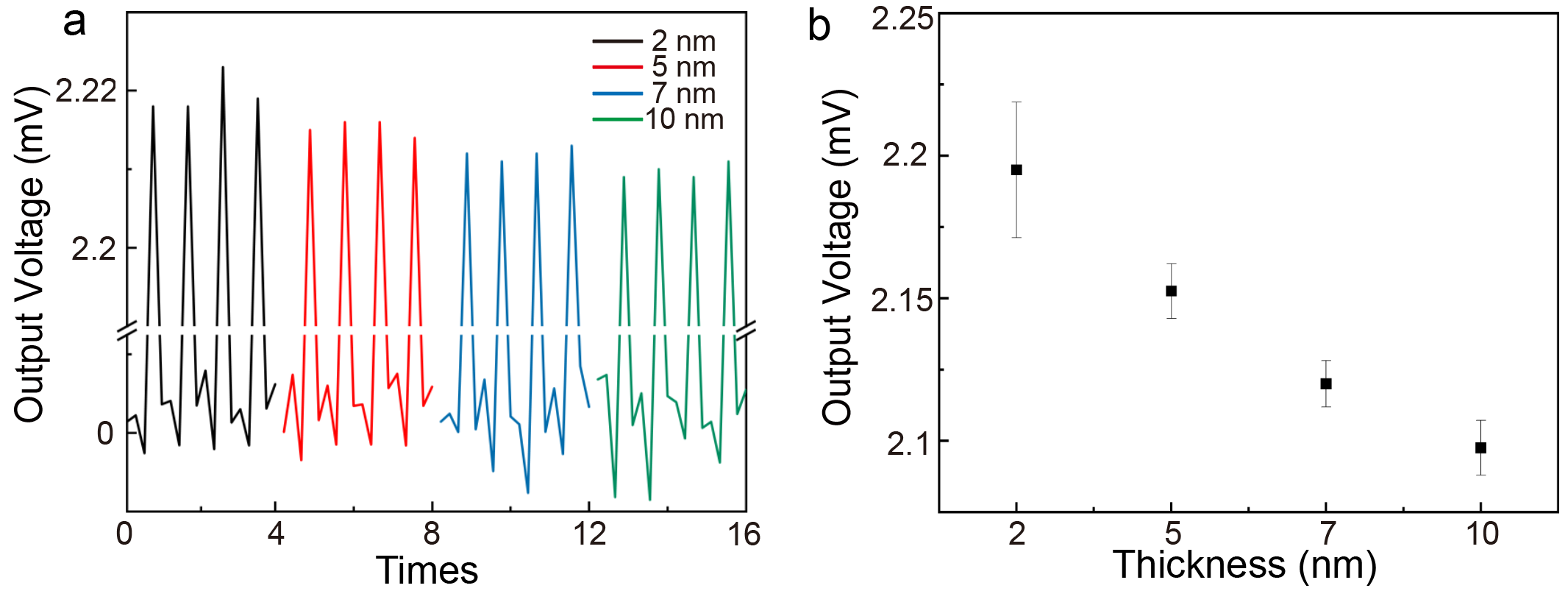


**Figure S14.** a) The output voltage of nano-stress sensors with different thicknesses of c-In_2_O_3_ under 100 nN. b) The c-In_2_O_3_ nano-stress sensor output voltage as a function of thicknesses under 100 nN force, in which results are extracted from four devices.


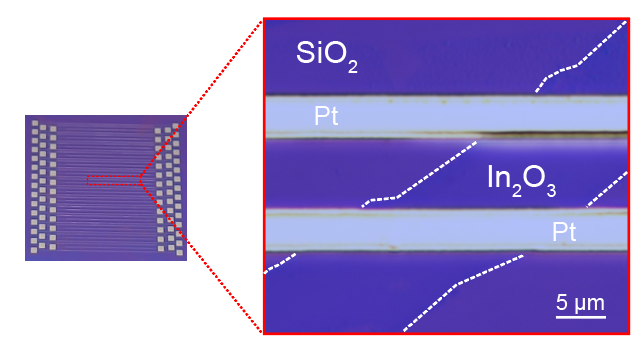


**Figure S15.** Optical images of the nano-sensor. The contact pads are 0.5 х 0.5 mm^2^, and dual Pt electrodes distance is 7 μm.


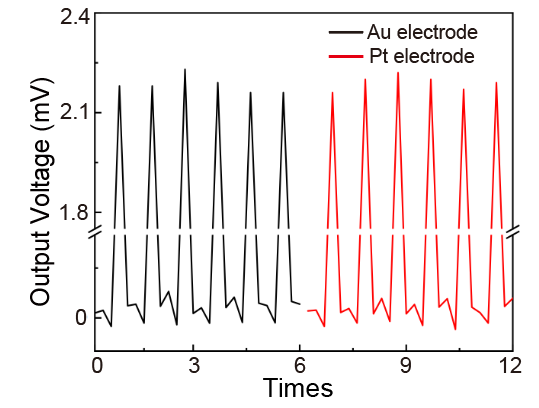


**Figure S16.** The output voltage of c-In_2_O_3_ nano-stress sensors with 2 nm thickness fabricated with Au and Pt electrodes under 100 nN force, which shows no significant difference.

**References**

[1] P. Hess, *Applied Surface Science* **1996**, *106*, 429.

[2] J. Bartolomé, P. Hidalgo, D. Maestre, A. Cremades, J. Piqueras, *Applied Physics Letters* **2014**, *104* (16).
